# The Impact of Physical Distancing and Associated Factors Towards Internet Addiction Among Adults in Indonesia During COVID-19 Pandemic: A Nationwide Web-Based Study

**DOI:** 10.3389/fpsyt.2020.580977

**Published:** 2020-09-03

**Authors:** Kristiana Siste, Enjeline Hanafi, Lee Thung Sen, Hans Christian, Adrian, Levina Putri Siswidiani, Albert Prabowo Limawan, Belinda Julivia Murtani, Christiany Suwartono

**Affiliations:** ^1^Department of Psychiatry, Faculty of Medicine, Universitas Indonesia – dr. Cipto Mangunkusumo General Hospital, Jakarta, Indonesia; ^2^Faculty of Psychology, Atma Jaya Catholic University, Jakarta, Indonesia

**Keywords:** Coronavirus Disease 2019, internet addiction, physical distancing, psychopathology, sleep quality, Indonesia

## Abstract

**Introduction:**

Physical distancing has encouraged the public to utilize the Internet for virtually all daily activities during the COVID-19 pandemic. This study aimed to assess the impact of COVID-19 on Internet addiction (IA) prevalence and analyzed the correlated factors during quarantine and pandemic.

**Methods:**

An online survey was generated, comprising of a sociodemographic section, Internet Addiction Diagnostic Questionnaire (KDAI), Symptoms Checklist-90, and Pittsburgh Sleep Quality Index. The hyperlink was disseminated through social media, companies, and universities. Overall, 4,734 adults, (mean age 31.84 ± 7.73 years old and 55.2% males) representing all 34 provinces of Indonesia, gave valid responses.

**Results:**

Point prevalence of IA during the COVID-19 pandemic was 14.4% in Indonesian adults. Online duration increased by 52% compared to before the pandemic. Physical distancing was not established as a risk of IA. Increased daily online duration, specific motivations, types of application, and having confirmed/suspected COVID-19 cases within the household were predictive of IA. All subscales of SCL-90 and PSQI were higher in the group with positive/suspect cases of COVID-19 within households and were correlated to higher scores of IA.

**Discussion:**

Physical distancing alone was not associated with an increased risk of IA. The prevalence of IA during COVID-19 was higher than the previously proposed rate among Indonesian adults, which might be related to digital activities associated with COVID-19 and the popularity of virtual socializing. Furthermore, psychopathologies and sleep disruptions were related to IA occurrences and especially prevalent in groups with proximity to COVID-19. Fear of COVID-19 contraction and rampant misinformation of COVID-19 probably contributed to these factors, which potentially harbor long-term consequences.

**Conclusion:**

The current study demonstrated a high point prevalence of IA and identified several preventable factors predictive of IA during home-quarantine and COVID-19, especially in adults with confirmed/suspected COVID-19 cases within the household. However, physical distancing did not increase the odds of IA. Public health agencies should maintain physical distancing advisory while providing adaptive psychiatric education and service.

## Introduction

The Coronavirus Disease 2019 (COVID-19) has grappled the world and presented a crisis of unprecedented magnitude. The effects are profound and far-reaching, not only on physical health but also mental health and social and financial repercussions. According to the World Health Organization (WHO), by the end of June 2020, there were more than 10 million confirmed COVID-19 cases and over 500,000 deaths worldwide (1). In Indonesia, there were about 55,000 confirmed cases, the highest in Southeast Asia, and nearly 3,000 deaths across the country as of late June 2020 (2). Though actual numbers could be much higher than that of the official reports as the testing capacity has not been brought up to speed in Indonesia (3).

To suppress further spread of COVID-19, WHO declared the importance of physical distancing by keeping a distance of at least 1 meter from each other, limit spending time in crowded places or groups, and wearing face masks (4). Concurrently, Indonesia recommended stay-at-home advice on March 15, 2020 and further implemented “large scale social restrictions”, locally known as PSBB (*Pembatasan Sosial Berskala Besar*), by April 10, in response to the soaring cases of COVID-19 nationally. During PSBB, public transport, travel and public places are either restricted or closed, people are encouraged to work or study from home, and large gatherings (e.g. marriages and religious affairs) are also prohibited—all in order to limit physical or direct social contact (5).

Due to this physical distancing policy, people turn to the Internet to perform their daily routines, from studying, meeting, performing a religious activity, and socializing. Utilization of the Internet also offered ease in disseminating public advice, delivering telehealth, and sharing of data between countries. At the same time, COVID-19 has intensified dependency on Internet and overloaded the public with barrages of false news and hoaxes—”an epidemic of misinformation”—leading to the menacing image of COVID-19 and propelling a climate of anxiety and panic (6). A study on nearly 60,000 respondents in China identified 35% of the general community to demonstrate psychological distress (7) and a separate longitudinal study indicated that the psychological symptoms persisted for at least a month (8). Afflicted by the heavy mental burden and deprived of their regular coping outputs, substantial proportion of people would turn to the Internet as their coping mechanism (9). Steam, a leading game distributor, reported more than 20 million concurrent active users, the highest number in its 16-year history (10). Gao et al., found that 82% of the Chinese samples were frequently exposed to social media during the pandemic (11) and Ni et al., noticed that a third of the samples spent at least 2 h online per day for social media and COVID-19 news (12). Information overload and extended social media exposure were previously reported to increase the susceptibility towards Internet addiction (IA), loosely defined as the compulsivity, preoccupation, or dependence on the Internet regardless of the specific activity that leads to impairment and distress (13, 14).

There had yet to be any data on the current physical distancing and behavioral patterns impact on IA in Indonesia. To bridge this gap, the present study aimed to examine the relationship of physical distancing policy during the COVID-19 pandemic to the prevalence of IA and associated effects of the psychological correlates among Indonesian adults. The current study aims to ensure psychological and physical well-being during and after the COVID-19 pandemic as well as future outbreaks. Moreover, the results can contribute to developing a national-scale regulation on Internet usage and guide public health measures.

## Methods

### Participants and Procedure

The authors devised an online survey using Google Form, beginning with an outline on the study’s purpose, respondents criteria, and management of data; then, each respondent was asked for informed consent to participate and an author’s email for correspondence was provided should queries arise. Those who did not provide consent to participate were directed to finish without answering the survey. The survey comprised of a sociodemographic section (e.g., gender, age, household income, occupations, and residence), quarantine elements (practice, location, confirmed/suspected cases within the household), and Internet usage characteristics (duration prior and during quarantine, age of first Internet usage, motives, and frequent social media applications or game genres), then followed by Internet Addiction Diagnostic Questionnaire (KDAI), Symptoms Checklist 90 (SCL-90), and Pittsburgh Sleep Quality Index (PSQI). Game genres were categorized into multiplayer online battle arena (MOBA), massively multiplayer online role playing games (MMORPG), first-person shooting (FPS), and casual games [defined as per prior study (15)], all were provided with examples. The survey in total spanned 18 pages and required about 45–55 min for completion, although duration could not be evaluated in Google Form to prevent reporting bias.

Physical distancing was defined as working/studying from home, alternating workday, and/or the physical distancing practices as per the guideline from Indonesian COVID-19 Response Acceleration Task Force (GTPP COVID-19) (16). Respondents were questioned whether they and/or any household member had been declared as COVID-19 suspect cases and/or diagnosed with COVID-19, following the descriptions provided by the GTPP COVID-19 (16), Indonesian Ministry of Health (17), and World Health Organization (18). Province of residence was categorized into whether PSBB had been implemented at the commencement of the study based on data from GTPP Covid-19 which encompassed DKI Jakarta, West Java, East Java, Central Java, Banten, West Kalimantan, North Kalimantan, Gorontalo, West Sumatera, Riau, and South Sulawesi (19). Income levels were determined based on classification by the World Bank (20).

A shortened hyperlink was generated and disseminated by the research team through social media and to the corporate secretaries of each Indonesian state-owned company and university academics between April 28 (44 days since stay-at-home notice and 18 days since PSBB) and June 1, 2020. Afterward, all respondents were suggested to pass on the survey link to others, employing a snowballing strategy. This was similar to the method adopted in a COVID-19 study among the Chinese general population (21). Enrolled respondents were (i) asked to provide emails (names were not requested) to prevent multiple responses; (ii) ≥21 years old; (iii) currently residing in Indonesia; (iv) and capable of understanding Bahasa Indonesia. Responses of non-consenting (*n* = 23), duplicates (*n* = 5), and currently not residing in Indonesia (*n* = 13) were removed. Identifying personal information (i.e., emails) were exclusively accessible to the research team. They were only inspected for duplicates and dropped before further data scrutiny; as such, the research team could not link the data and participant. Overall, a total of 4,734 respondents completed the survey encompassing all 34 provinces and seven islands (Java 62.7%, Sumatera 18.3%, Kalimantan 8.6%, Sulawesi 5.8%, Nusa Tenggara 2.7%, Papua 1.7%, and Maluku 0.3%) across Indonesia. The survey was part of a larger study simultaneously targeting adolescents, and 150 adult respondents mistakenly answered the Pediatric Symptoms Checklist 17 instead of SCL-90, their responses were omitted during analysis (*n* = 4,584).

### Instruments

#### Internet Addiction Diagnostic Questionnaire (KDAI)

KDAI (22) was developed in Indonesia with excellent reliability (α = 0.942), sensitivity (91.8%), and negative likelihood ratio (0.11). The tool is self-administered, and consists of 7 subscales, namely, withdrawal (e.g. “I feel very disturbed if forced to stop using the Internet”, 8 items), loss of control (e.g. “I forgot about time when I am on the Internet”, 9 items), priority enhancement (e.g. “I cut back on doing other fun activities so I could be on the Internet”, 6 items), negative consequences [e.g. “My tasks are neglected (such as homework, etc.) because I use the Internet too much”, 7 items], mood modification (e.g. “My life feels more comfortable when I am on the Internet”, 5 items), salience (e.g. “I keep on thinking of using the Internet even though I am currently doing other tasks”, 6 items), and impairment (e.g. “I tried to limit my time on the Internet, but I failed”, 3 items). Each statement is scored with a 7-point Likert scale, 0 (= not applicable), 1 (= very rarely) to 6 (= always). A score of ≥108 indicates IA (out of 264 maximum). The reliability of domains was satisfactory, Cronbach’s alphas range from 0.641–0.933, and overall α = 0.979.

#### Symptoms Checklist 90 (SCL-90)

SCL-90 is a self-reported tool to assess psychopathological symptoms, namely: somatization, obsessive-compulsiveness, interpersonal sensitivity, depression, anxiety, phobic anxiety, hostility, paranoid ideation, psychoticism, an additional domain, and overall global symptom index (23, 24). The instrument has 90 statements scored on a 5-point Likert scale, 0 (= Never) and 4 (= Always), within the last one month. SCL-90 had been translated to Bahasa Indonesia with good validity 82.9% sensitivity and 83.0% specificity (25). Subscales consistencies were acceptable, with α ranging 0.837–0.987.

#### Pittsburgh Sleep Quality Index (PSQI)

The PSQI (26) is a widely utilized tool to assess sleep quality on clinical or non-clinical populations (27), the reliability in this study was α=0.845. The questionnaire has 24 items, of which 20 are multiple choices and another 4 open-ended questions. Furthermore, 5 items require the assessment of a partner or another individual on the sleeping pattern of the subject. The 19 self-answered questions on PSQI can be pooled into 7 components and each weighted between 0–3 (maximum 21), scores >5 indicate poor sleep quality. The Indonesian version of PSQI was validated with reliability of α=0.79, content validity 0.89, and specificity of 81% (28).

### Data Analysis

Descriptive analyses were performed for all data, general characteristics were stratified by gender, and key sociodemographics were scrutinized against IA using logistic regression. Age was dichotomized into 21–40 and >40 years old in reference to the definition by Indonesian Association of Pediatrics (29) of adolescents as those aged 10- to 20- years old and early adulthood within developmental psychiatry perspective (30) considered as between 20 and 40 years old. The age of first Internet use was adopted from another study’s observation (31) and was noted to be a significant predictor in a prior dissertation study among Indonesian adolescents (22). Duration of Internet use was categorized based on a previous research (32) definition of excessive Internet usage (>5 h) and current median of data at 10 h. Lastly, number of social media use was determined based on data median of 3. Correlations matrix between KDAI, SCL-90, and PSQI was generated by Spearman’s (*rho*) correlation as data had non-normal distributions. Bootstrapping was also performed for correlation analyses and set at 5,000 samples. All statistical tests were performed on SPSS 23.0 for Windows (IBM, USA). Data were deemed significant if *p <*0.05 and 95% confidence interval (CI) provided where appropriate.

### Ethics

The study was approved by the Institutional Ethics Committee of Faculty of Medicine, Universitas Indonesia—dr. Cipto Mangunkusumo General Hospital (Ref: KET-413/UN2.F1/ETIK/PPM/00/02/2020). Informed consent was required for all respondents.

## Results

### Sociodemographic Profile

Characteristics of the study’s subjects are presented in **Supplementary Table 1**. Over half of the samples were males (N = 2,612; 55.2%). Mean age of subjects was 31.84 ± 7.733 (Range = 21–69), and on average males were older than females. Males (Onset age = 17.78 ± 6.598) also tended to adopt the Internet later compared to female (15.92 ± 5.524). Most of our subjects had attained higher education (*N* = 3590; 75.8%) and are in the workforce as office workers/proprietors (*N* = 3627; 76.6%). Vast proportion of our population was already married (*N* = 2995; 63.3%). Majority of subjects (47.6%) were within the middle-upper SES bracket. About 66.8% of the subjects reported living in provinces that had not implemented the PSBB. Around 187 (3.95%) respondents acknowledged having confirmed/suspected COVID-19 cases within their households and 22.5% of them were classified as Internet addicts.

Internet usage behaviors of participants were also evaluated before and during COVID-19 pandemic. Most subjects (79.95%) perceived to have increased Internet duration during the COVID-19 pandemic and both female and male on average had an increase of 3.43 h per day comparing usages before and during COVID-19 pandemic. Amid the pandemic, 25.4% of respondents utilized the Internet for 0–5 h per day, 34.2% for 6–10 h daily, and 40.3% for ≥11 h. Almost all subjects (97.8%) first used the Internet when they were older than 8 years old. Monthly Internet expenditure among respondents was mainly over 250,000 IDR (17.72 USD at conversion rate of 14,100). Handphone was the most preferred gadget (96.2%) for accessing Internet, followed by PC/Laptop (57.8%). Main motives for using the Internet were academic/occupation-related (39.5%), social media (31.7%), seeking information (20.4%), entertainment (video, music, or reading; 5.9%), online games (1.8%), online shopping (0.4%), online pornography (0.1%), cyber-relationship (0.1%), and none for online gambling. Most frequent social media used in the study sample were *WhatsApp* (95.0%), *Instagram* (81.9%), *Facebook* (55.4%), *Telegram* (29.8%), *Twitter* (29.1%), *Line* (23.3%), *TikTok* (8.7%), and the least was *WeChat* at 1.4%. Overall, 41.8% of respondents used 4 or more social media applications. Of the respondents that play online games (47.6%), 31.0% preferred casual games, 14.1% MOBA, 2.3% MMORPG, and 0.23% FPS.

### Internet Addiction and Correlated Characteristic Factors

Point prevalence of IA during COVID-19 pandemic among Indonesian adults was 14.4% (95% CI 13.4–15.5%). Bivariate analyses (See **Supplementary Table 2**) were conducted to several related factors with IA as the dependent variable. Significant variables on bivariate analysis and variables deemed potentially predictive based on past studies were included into multivariate analysis.

Multivariate analysis (**Table 1**) showed that several variables were related to IA. Group having COVID-19 confirmed/suspected cases within household had significantly higher risk to IA. Changes of Internet duration, particularly, increased online duration ≥11 h were predictive of IA. Several motives of digital activities (social media, online gaming, information seeking, and entertainment) also augmented the odds of IA. Particular social media applications (*Twitter* and *LINE*) and certain type of online games (casual games and MOBA games) were found significantly associated to IA.

**Table 1:** Multivariate analysis of variables related to Internet addiction.

Variables	B	SE	Wald	df	AOR (95% CI)
**Gender (ref: Female)**					
Male	-0.09	0.099	0.822	1	0.914 (0.752–1.110)
**Age (ref: >40)**					
21–40	0.321	0.167	3.695	1	1.379 (0.994–1.914)
**Age of First Internet Use (ref: >8)**					
≤ 8	0.384	0.258	2.216	1	1.468 (0.886–2.434)
**COVID-19 confirmed/suspected cases within household? (ref: No)**					
Yes	0.47	0.191	6.029	1	1.600* (1.099–2.328)
**Physical distancing (ref: No)**					
Yes	0.04	0.103	0.152	1	1.041 (0.850–1.275)
**Perceived Internet Duration Change (ref: Unchanged)**					
Increased	0.512	0.134	14.582	1	1.669*** (1.283–2.171)
Decreased	0.097	0.492	0.039	1	1.102 (0.420–2.891)
**Duration of Internet use during COVID-19 (ref: 0–5 h)**					
6–10	0.17	0.13	1.701	1	1.185 (0.918–1.531)
≥11	0.506	0.125	16.328	1	1.658*** (1.298–2.119)
**Main Motives of Internet use (ref: Academic/occupational)**					
Social Media	0.411	0.109	14.248	1	1.508*** (1.218–1.866)
Online Gaming	0.682	0.287	5.65	1	1.977* (1.127–3.469)
Blogging	-18.81	40192.97	0	1	–
Information Seeking	0.335	0.125	7.207	1	1.398** (1.095–1.785)
Online Shopping	-18.799	8814.669	0	1	–
Entertainment	0.484	0.18	7.187	1	1.622** (1.139–2.311)
Cyber-relation	1.318	1.24	1.129	1	3.736 (0.329–42.451)
Pornography	0.256	1.133	0.051	1	1.292 (0.140–11.904)
**Number of social media used**					
**(ref: ≥ 4)**					
0–3	-0.086	0.15	0.331	1	0.917 (0.684–1.230)
**Social Media Apps used**					
**(ref: Do not use)**					
Facebook	-0.042	0.101	0.169	1	0.959 (0.786–1.170)
Instagram	-0.12	0.136	0.781	1	0.887 (0.680–1.157)
Twitter	0.355	0.108	10.843	1	1.426*** (1.155–1.762)
LINE	0.239	0.115	4.273	1	1.270* (1.012–1.592)
WhatsApp	-0.33	0.203	2.643	1	0.719 (0.483–1.070)
TikTok	0.017	0.151	0.013	1	1.018 (0.757–1.369)
WeChat	0.082	0.391	0.044	1	1.085 (0.504–2.335)
Telegram	0.129	0.108	1.435	1	1.138 (0.921–1.407)
**Frequent Game Genres**					
**(ref: Do not play games)**					
MMORPG	0.277	0.274	1.022	1	1.319 (0.771–2.258)
MOBA	0.456	0.131	12.057	1	1.578*** (1.220–2.042)
FPS	0.919	0.709	1.679	1	2.507 (0.624–10.069)
Casual Games	0.217	0.102	4.55	1	1.243* (1.018–1.517)
**Constant**	**-2.978**	**0.35**	**72.319**	**1**	0.051***
**-2LL**	**3,545.79**				
**Nagelkerke R2**	**0.067**				
**Hosmer-Lemeshow**	**p = .618**				
**Model χ2 = 175.037, df = 30, p <.001**					

*p < .05; **p ≤ .01; ***p ≤ .001.

### Internet Addiction, SCL-90, and PSQI

Comparing scores of participants with COVID-19 confirmed/suspected cases within their households and without, the former scored on average higher across all subscales of SCL-90 and PSQI, which were statistically significant, *p <*0.001 (**Table 2**). Depression (9.02 ± 11.46 vs. 5.43 ± 8.06), obsessive-compulsive (7.06 ± 7.80 vs. 4.78 ± 6.17), somatization (6.83 ± 9.04 vs. 4.61 ± 6.69), and interpersonal sensitivity (6.49 ± 8.16 vs. 4.08 ± 5.86) were among the subscales with largest difference between the two groups.

**Table 2:** SCL-90 and PSQI profiles of respondents diagnosed as suspected cases or having COVID-19 confirmed cases within a household.

Variable^a^	COVID-19 Confirmed or Suspected Case		*Z^b^*
	Yes (n = 178)	No (n = 4406)	
**GSI**	56.70 ± 67.26	36.45 ± 49.35	4.863***
	30.5 (9.0,82.5)	17.0 (3.0,50.0)	
**Depression**	9.02 ± 11.46	5.43 ± 8.06	4.339***
	4.0 (1.0,13.0)	2.0 (0.0,7.0)	
**Anxiety**	5.16 ± 7.58	3.24 ± 5.43	4.091***
	2.0 (1.0,6.0)	1.0 (0.0,4.0)	
**Obsessive-Compulsive**	7.06 ± 7.80	4.78 ± 6.17	4.447***
	4.5 (1.0,10.0)	2.0 (0.0,7.0)	
**Phobic Anxiety**	3.97 ± 5.54	2.46 ± 3.89	4.812***
	2.0 (1.0,5.0)	1.0 (0.0,3.0)	
**Somatization**	6.83 ± 9.04	4.61 ± 6.69	4.025***
	3.0 (1.0,9.0)	2.0 (0.0,6.0)	
**Interpersonal Sensitivity**	6.49 ± 8.16	4.08 ± 5.86	4.218***
	3.0 (0.0,9.0)	2.0 (0.0,6.0)	
**Hostility**	2.88 ± 3.94	1.81 ± 3.02	4.885***
	1.0 (0.0,4.0)	1.0 (0.0,2.0)	
**Paranoid**	3.80 ± 5.22	2.49 ± 3.99	3.709***
	1.0 (0.0,6.0)	1.0 (0.0,4.0)	
**Psychoticism**	5.08 ± 7.12	3.32 ± 5.52	3.756***
	2.0 (0.0,8.0)	1.0 (0.0,4.0)	
**Additonal**	5.51 ± 5.64	3.67 ± 4.57	4.936***
	4.0 (1.0,8.0)	2.0 (0.0,6.0)	
**PSQI**	6.61 ± 3.31	5.46 ± 3.08	4.508***
	6.0 (4.0,9.0)	5.0 (3.0,7.0)	

^a^Data presented as Mean ± SD and Median (IQR); GSI, Global Severity Index; PSQI, Pittsburgh Sleep Quality Index; ^b^Mann-Whitney U test; ***p ≤ .001.

Mean of the SCL-90 Global Severity Index (GSI) score was 37.24 ± 50.3 and respondents scored 5.53 ± 3.10 on average for PSQI. Other domains of SCL-90 ranged from 1.85 to 5.57 with depression having the highest score (5.57 ± 8.24). IA was correlated to the GSI and all subscales of SCL-90 positively with range of *r* = .249 to.320 (*p* < 0.001); moreover, higher score of KDAI was also correlated with higher score PSQI, *r = *.225 (*p* < 0.001). Detailed correlation matrix is shown in **Table 3**.

**Table 3:** Correlation matrix analysis between KDAI score, sub-scales of Indonesian Symptoms Checklist 90, and PSQI.

	1	2	3	4	5	6	7	8	9	10	11	12	13
**1. KDAI**	–												
**2. GSI**	0.313**	–											
**3. Depression**	0.303**	0.927**	–										
**4. Anxiety**	0.303**	0.865**	0.787**	–									
**5. Obsessive-Compulsive**	0.311**	0.930**	0.858**	0.781**	–								
**6. Phobic Anxiety**	0.256**	0.805**	0.760**	0.690**	0.737**	–							
**7. Somatization**	0.249**	0.858**	0.752**	0.845**	0.773**	0.678**	–						
**8. Interpersonal Sensitivity**	0.313**	0.911**	0.850**	0.760**	0.848**	0.705**	0.721**	–					
**9. Hostility**	0.301**	0.823**	0.762**	0.705**	0.773**	0.645**	0.679**	0.795**	–				
**10. Paranoid**	0.302**	0.570**	0.800**	0.729**	0.803**	0.677**	0.684**	0.856**	0.783**	–			
**11. Psychoticism**	0.320**	0.872**	0.820**	0.753**	0.816**	0.694**	0.704**	0.828**	0.752**	0.823**	–		
**12. Additional**	0.284**	0.902**	0.814**	0.759**	0.828**	0.711**	0.763**	0.802**	0.724**	0.754**	0.793**	–	
**13. PSQI**	0.225**	0.538**	0.493**	0.477**	0.505**	0.397**	0.485**	0.482**	0.442**	0.442**	0.460**	0.534**	–
**Mean**	66.51	37.24	5.57	3.32	4.87	2.52	4.69	4.18	1.85	2.54	3.39	3.74	5.53
**SD**	41.55	50.30	8.24	5.54	6.26	3.97	6.81	5.98	3.06	4.05	5.60	4.63	3.10
**Median**	62	17	2	1	3	1	2	2	1	1	1	2	5
**IQR**	45	48	8	4	7	4	6	6	2	4	5	6	5

**p < 0.01; All 95% CIs of the correlation coefficients were above 0 through bootstrapping (5,000 iterations).

## Discussion

The present study indicated substantial IA point prevalence (14.4%) among Indonesian adults during the COVID-19 pandemic. This is, as far as the authors are aware, the first nationwide study on IA in Indonesia. A previous study on Indonesian university students (20.9 ± 2.52 years old) proposed a rate of 3.2% for IA (33) and another study measuring Internet Gaming Disorder found 3.0% prevalence (34). To note, there was a difference of instruments utilized and subject demographics to the current study. During COVID-19 pandemic, Priego-parra et al., reported 10.2 and 0.2% of moderate and severe IA, respectively, among Mexicans (35) and Sun et al. demonstrated a rate of 4.3% of severe IA in China (36).

In this study, only a third of respondents were living in provinces enforcing PSBB, yet more than 70% practiced physical distancing; this is reasonable as the virus had spread nationally and stay-at-home notice was issued across the country. Recent studies have demonstrated increases in symptoms of post-traumatic distress, anxiety, depression, and physical symptoms during COVID-19 self-quarantine (7, 8). However, the current study found that the sole action of physical distancing was not a predictor of IA. It is assumed that the availability of multiple channels for maintaining social connections (37) and public education on self-management during isolation (38, 39) has dampened the risk posed by physical distancing to a certain extent. Furthermore, the methods of said physical distancing and the degree of altered routines were also variable between individuals since Indonesia did not enter a mandatory “lockdown”.

The psychological disturbances were considerable in our study with respondents scoring highly in all subscales of SCL-90 (25). Moreover, the group with confirmed/suspected COVID-19 case within their households scored higher compared to the ordinary population—particularly subscales of depression, obsessive-compulsiveness, somatization, and interpersonal sensitivity. Subsequently, a significant correlation between having confirmed/suspected COVID-19 cases within household and IA was observed in this study, AOR = 1.600 (95% CI = 1.099–2.328), and our data also demonstrated significant correlations of IA with all subscales of SCL-90 (*r* = 0.249 to 0.320). Other studies in Spain (40, 41) and Japan (42) had also linked promixity or close contacts toward COVID-19 positive and suspect cases with increased psychological distress; although as far as the authors are aware this study is the first to demonstrate a linkage to IA.

The current and several other studies indicated that COVID-19 fear and prolonged quarantine period might have driven people to experience depressive and anxiety symptoms (7, 8, 35, 43, 44). Recreational online activities are often a mechanism to cope with anxiety and alleviate depressed mood (9). However, abusive usages may actually exacerbate anxiety and depression and reinforce the compulsion to use the Internet, developing a maladaptive coping mechanism (9, 10). PSBB encouraged people to utilize the Internet for virtually all facets of daily activities, thus exponentially increasing their Internet exposure. Our study revealed that there was a significant increase of duration of Internet usage of about 52% during COVID-19 and nearly all respondents utilized mobile phones for accessing the Internet. This finding was in line with Indonesian communication providers reports of rising broadband traffic during the pandemic (45, 46).

Additionally, this study also found that being online for over 11 h per day posed significant risk for IA. Past studies have mentioned the bidirectional relationship of time spent online and IA (47, 48). Internet duration as defined in our study, was irrespective of the specific digital activities or purposes. Therefore, further studies are required to stratify risks with respect to distinction of durations.

Apart from online duration, particular predominant motivations were found to be also related to IA. Social media and online gaming were two types of specific IA (49). Our findings affirmed association between social media, gaming, and IA. Despite their various features, all social media [e.g. *Instagram* (50), *Facebook* (51, 52), *WhatsApp* (53), *LINE* (54)] comprehensibly elicit some IA risk. The current study revealed that in our population, *Twitter* were correlated to higher odds of IA. This result could be explained by the fact that Indonesia has an enormous active *Twitter* userbase (55). In the current pandemic, Indonesia was also the second-highest based on the number of posts regarding COVID-19 topic on *Twitter* among Asia-Pacific countries (56). Social media use cannot be separated from information-seeking behavior. Motives of Internet use for information seeking was also related to IA in this research contrasting another study, which suggested no association (57). Keeping in mind, this study examined the behavior amidst the COVID-19 pandemic and people tend to desire and seek excessive information to stay updated during times of crisis (58–60). Improper information regulation regarding COVID-19 might enhance information overload, psychological stress, and risk of IA (9, 12, 59, 61).

On the analysis of psychopathology among the respondents, this study revealed that those with confirmed/suspected cases of COVID-19 within household scored almost twice as high than their counterpart in subscales of obsessive-compulsive, interpersonal sensitivity, somatization, and psychoticism. The severity of obsessive-compulsive traits which could be motivated through the thoughts of a heightened risk of coronavirus contraction leading to frequent hand-washing and other preventive measures (62), that would be reasonably heightened in individuals with confirmed/suspected COVID-19 cases within their households. Obsessive-compulsiveness tendencies are more likely to occur in Internet addicts than non-addicts. Since this group is intrusively preoccupied with the Internet, required longer timespan online, and experienced withdrawal when trying to reduce their digital life (63). Similarly, recommendations to maintain distance and avoid public transportations and gatherings might spur phobic anxiety and interpersonal sensitivity symptoms (7).

Intriguingly, somatization, and psychoticism were also considerably higher in the group with confirmed/suspected COVID-19 cases within their household and correlated to higher scores of IA. Illusive physical symptoms, observed even among the public during COVID-19 (7, 8), could be magnified among those with IA mediated partially by sleep disruptions (64, 65) and importantly, Internet addicts seemingly expressed depression as somatic manifestations (66, 67). Multiple brief psychotic cases of previously healthy individuals and absent psychiatric history had been reported as well in relation to COVID-19 (68, 69), that might be attributed to “coronaphobia” (70), the irrational fear and impression of helplessness and impending death due to exaggerated misinformation of COVID-19 (6, 71). Biologically, psychotic episodes had also been associated in people with seroreactivity to previous coronaviruses with possibility of neurotropism (72) or inflammatory damage (8). A prospective study described the persistence of problematic Internet use and frequent non-clinical psychotic events (73) and these Internet addicts were prone towards psychoticism-extraversion-neuroticism and instability in impulse control (74, 75).

Likewise, a particular game genre, i.e., MOBA, is related to IA *via* impulsivity as the key factor (76). Additionally, MOBA is growingly regarded as the more popular genre among amateur and professional gamers (76–78). Our study found that MOBA was related to IA during COVID-19 outbreak. Interestingly, mobile data use for Mobile Legend, a MOBA game currently sensational in Indonesia, has been reported to escalate during the home quarantine period (79). Other types of games, e.g. MMORPG (80) and FPS (81), are also proposed to raise the susceptibility towards IA. These might not be correlated to IA within our data due to the much older demographic, less availability of such genre in mobile devices, and decreasing popularity (76, 82). Subsequently, the present study discovered entertainment intent (e.g., watching a video, listening to the music, or reading comics/novels) to be predictive of IA during this pandemic. Binge-watching can be recognized as an abusive behavior, and the Internet reinforced the behavior through offering myriads of choices, personalized recommendations, autoplay, and socializing (e.g. comment sections and fandoms) which proliferate the addictive nature (83, 84), particularly in the times of reduced physical socializing amid COVID-19 and people turning to streaming services (9, 46).

The results of sleep quality in this study resonated with other COVID-19 studies, where fear of contracting the virus and isolation reduced sleep quality (85–87); the effect is more pronounced in subjects who had COVID-19 confirmed/suspected cases within household, as they scored higher in PSQI compared to those who reported no cases within their household. A study on COVID-19 patients uncovered insomnia as the second most diagnosed neuropsychiatric disorder (88). However, acknowledging the pervasive effects of COVID-19, other causes of worries should be recognized, such as social stigma, financial disturbances, and adversity in accessing basic needs (85). The current study established a positive correlation between scores of KDAI and PSQI (*r* = 0.225), indicating that apart from fear of contracting COVID-19, sleep disturbance was also related to problematic Internet use. A meta-analysis, with a majority of studies originating from Asia, asserted that Internet addicts had longer sleep latency, shorter adequate sleep time, and lower sleeping efficiency compared to their counterparts (89), in part due to the drive and preoccupation to Internet usage as well as potential inhibition of melatonin secretion due to the screens’ blue-lights (90). Extensive Internet leisure activities (e.g., social media, online gaming, shopping, and gambling) had also been highlighted to curtail sleep duration (91), specifically within the period before bed (92). This translated to subjective lack of sleep quality, excessive daytime sleepiness, poor daytime functioning, and diminished self-control. Sleep deprivation is also linked to physical complaints, depression, anxiety, and suicidal tendencies (93), exacerbating the relationship between sleep quality and IA through psychological correlates. Emerging evidences pointed to the possibility of chronic neuropsychiatric sequalae (sleep disturbances and psychosis) among COVID-19 patients (88, 94) and past study highlighted sleep disturbances (60) and psychosis (72) were observed even in recovered cases of previous coronaviruses. Thus, more long-term observations will be required to astutely assess the correlation of IA, sleep perturbation, and psychotic tendencies.

The study inherently had several limitations, firstly, with its online survey methods certain respondent and reporting biases existed and the study was not be able to reach those without Internet connections. The study employed total sampling, which is inferior to random sampling. There was also an overrepresentation of the higher income bracket and particular occupational sector (office workers/proprietors), which could lead to selection bias. Self-reported instruments would also deposit additional biases, such as social desirability. The causal relationships between IA and correlates could not be established within this study due to the transversal nature.

Nonetheless, this study was the first nationwide study of IA in Indonesia and during the COVID-19 pandemic. The sample size and geographical spread were adequate to explore correlations and interactions to provide substantial evidence for national guidance. The data of this study could also be used as a comparison for future prospective studies in Indonesia.

## Conclusion

The current study identified the rate of IA at 14.4% among the adult Indonesian population during the COVID-19 pandemic and home-isolation period. Extensive Internet duration, specific Internet motives, psychopathologies, and decreased sleeping quality were found to be correlated to IA during this pandemic, especially in group with confirmed/suspected COVID-19 cases within household. However, the act of physical distancing was not shown to increase the risk of IA. In light of these, public health bodies must maintain physical distancing recommendations and other public health measures, while consolidating and promoting mental health literacy, psychological warning signs, and adaptive psychiatric services during this tumultuous time.

## Data Availability Statement

The raw data supporting the conclusions of this article will be made available by the authors, without undue reservation.

## Ethics Statement

The studies involving human participants were reviewed and approved by Institutional Ethics Committee of Faculty of Medicine, Universitas Indonesia—dr. Cipto Mangunkusumo General Hospital. The patients/participants provided their written informed consent to participate in this study.

## Author Contributions

KS and EH conceived, designed, and supervised the study. KS, EH, LS, HC, and AL contributed data or analysis tools. KS, EH, LS, HC, AL, A, LS, and BM collected the data. KS, EH, LS, HC, and CS performed the data analysis. KS, EH, LS, HC, AL, A, LS, and BM wrote the manuscript. KS and CS secured funding for the study. All authors contributed to the article and approved the submitted version.

## Funding

This study received funding from the Ministry of Research and Technology/Centre of National Research and Innovation of Republic of Indonesia through the “*Konsorsium Riset dan Inovasi Untuk Percepatan Penanganan Corona Virus Disease 2019 (Covid-19)*” (Ref.: 106/FI/PKS-KCOVID-19.F/VI/2020). The funders had no role in the design, data collection, analysis and interpretation of data, write-up, and/or publication of this study.

## Conflict of Interest

The authors declare that the research was conducted in the absence of any commercial or financial relationships that could be construed as a potential conflict of interest.

## Abbreviations

PSBB, Large-scale social distancing; GTTP COVID-19, Indonesian COVID-19 Response Acceleration Task Force.
