# Supplementary material for: The Impact of Physical Distancing and Associated Factors Towards Internet Addiction Among Adults in Indonesia During COVID-19 Pandemic: A Nationwide Web-Based Study
Source: Front Psychiatry. 2020 Sep 3;11:580977. doi: 10.3389/fpsyt.2020.580977 (PMC7495250; doi:10.3389/fpsyt.2020.580977)
Supplement: Supplementary file 1 [file Table_1.docx]

Supplementary Material:

Table 1. Sociodemographic profile of respondents

| **Variables** | **Male (N=2612)** | | **Female (N=2122)** | | **Overall (N=4734)** | |
| --- | --- | --- | --- | --- | --- | --- |
|  | **Mean** | **SD** | **Mean** | **SD** | **Mean** | **SD** |
| **Age** | 33.01 | 7.801 | 30.39 | 7.401 | 31.84 | 7.733 |
| **Age of First Internet Use** | 17.78 | 6.598 | 15.92 | 5.524 | 16.94 | 6.209 |
| **Daily Internet Duration During COVID-19** | 9.52 | 5.613 | 10.64 | 5.724 | 10.02 | 5.689 |
| **Daily Internet Duration Prior COVID-19** | 6.35 | 4.593 | 6.89 | 4.566 | 6.59 | 4.588 |
|  | **n** | **%** | **n** | **%** | **n** | **%** |
| **Education** |  |  |  |  |  |  |
| Up to Senior High School | 480 | 18.3 | 198 | 9.3 | 678 | 14.3 |
| Diploma | 247 | 9.5 | 219 | 10.3 | 466 | 9.8 |
| Higher Education | 1885 | 72.2 | 1705 | 80.3 | 3590 | 75.8 |
| **Occupation** |  |  |  |  |  |  |
| Students | 89 | 3.4 | 215 | 10.1 | 304 | 6.4 |
| Professionals | 107 | 4.1 | 256 | 12.1 | 363 | 7.7 |
| Office Workers/Proprietors | 2299 | 88 | 1328 | 62.6 | 3627 | 76.6 |
| Civil Servants | 109 | 4.2 | 206 | 9.7 | 315 | 6.7 |
| Currently Unemployed | 8 | 0.3 | 117 | 5.5 | 125 | 2.6 |
| **Marital Status** |  |  |  |  |  |  |
| Single | 767 | 29.4 | 972 | 45.8 | 1739 | 36.7 |
| Married/Divorced | 1150 | 70.6 | 1845 | 54.2 | 2995 | 63.3 |
| **Income** |  |  |  |  |  |  |
| Low | 103 | 3.9 | 97 | 4.6 | 200 | 4.2 |
| Lower-Middle | 847 | 32.4 | 845 | 39.8 | 1692 | 35.7 |
| Middle-Upper | 1339 | 51.3 | 914 | 43.1 | 2253 | 47.6 |
| Upper | 323 | 12.4 | 266 | 12.5 | 589 | 12.4 |
| **Province of Residence** |  |  |  |  |  |  |
| Enact PSBB^a^ | 765 | 29.3 | 805 | 37.9 | 1570 | 33.2 |
| Not Yet Enact PSBB^a^ | 1847 | 70.7 | 1317 | 62.1 | 3164 | 66.8 |
| **No. of Adults Living Together** |  |  |  |  |  |  |
| 0 | 281 | 10.8 | 195 | 9.2 | 476 | 10.1 |
| 1-2 | 1266 | 48.5 | 758 | 35.7 | 2024 | 42.8 |
| 3-5 | 882 | 33.8 | 990 | 46.7 | 1872 | 39.5 |
| >5 | 183 | 7 | 179 | 8.4 | 362 | 7.6 |
| **No. of Children Living Together** |  |  |  |  |  |  |
| 0 | 971 | 37.2 | 996 | 46.9 | 1967 | 41.6 |
| 1-2 | 1322 | 50.6 | 943 | 44.4 | 2265 | 47.8 |
| 3-5 | 302 | 11.6 | 178 | 8.4 | 480 | 10.1 |
| >5 | 17 | 0.7 | 5 | 0.2 | 22 | 0.5 |
| **Perceived Internet Change During COVID-19** |  |  |  |  |  |  |
| Increased | 2034 | 77.9 | 1751 | 82.5 | 3785 | 80 |
| Decreased | 42 | 1.6 | 16 | 0.8 | 58 | 1.2 |
| Unchanged | 536 | 20.5 | 355 | 20.5 | 891 | 18.8 |
| **Age of First Internet Use** |  |  |  |  |  |  |
| ≤8 | 58 | 2.2 | 44 | 2.1 | 102 | 2.2 |
| >8 | 2554 | 97.8 | 2078 | 97.9 | 4632 | 97.8 |
| **Internet Expenditure** |  |  |  |  |  |  |
| ≤250.000 IDR/17.72 USD | 36 | 1.4 | 94 | 4.4 | 130 | 2.7 |
| >250.000 IDR/17.72 USD | 2576 | 98.6 | 2028 | 95.6 | 4604 | 97.3 |
| **Motives of Using Social Media** |  |  |  |  |  |  |
| Making/Maintaining Friends | 314 | 12 | 287 | 13.5 | 601 | 12.7 |
| Posting Photos | 26 | 1 | 37 | 1.7 | 63 | 1.3 |
| Gaining Popularity/Attention | 10 | 0.4 | 5 | 0.2 | 15 | 0.3 |
| Information Seeking | 1923 | 73.6 | 1374 | 41.7 | 3297 | 69.6 |
| Stalking | 3 | 0.1 | 8 | 0.4 | 11 | 0.2 |
| Staying Updated with Trend | 53 | 2 | 46 | 2.2 | 99 | 2.1 |
| Pastime | 196 | 7.5 | 304 | 14.3 | 500 | 10.6 |
| Online Business | 3 | 0.1 | 11 | 0.5 | 14 | 0.3 |
| Do not Use Social Media or None of the Above | 84 | 3.2 | 50 | 2.4 | 134 | 2.8 |
| **Motives for Playing Online Game** |  |  |  |  |  |  |
| Fun and Exciting | 296 | 11.3 | 133 | 6.3 | 429 | 9.1 |
| For competing with other | 167 | 6.4 | 23 | 1.1 | 190 | 4 |
| Building friendship | 83 | 3.2 | 9 | 0.4 | 92 | 1.9 |
| Forming my ideal life/self | 24 | 0.9 | 28 | 1.3 | 52 | 1.1 |
| Relaxing and joyous | 574 | 22 | 438 | 20.6 | 1012 | 21.4 |
| To escape from reality | 289 | 11.1 | 230 | 10.8 | 519 | 11 |
| Do not play games or None of the above | 1179 | 45.1 | 1261 | 59.4 | 2440 | 51.5 |
| **Gadget** |  |  |  |  |  |  |
| Handphone | 2486 | 95.2 | 2066 | 97.4 | 4552 | 96.2 |
| PC/Laptop | 1467 | 56.2 | 1269 | 59.8 | 2736 | 57.8 |
| Tablet | 237 | 9.1 | 219 | 10.3 | 456 | 9.6 |
| Gaming Console | 8 | 0.3 | 2 | 0.1 | 10 | 0.2 |
| Smart TV | 38 | 1.5 | 44 | 2.1 | 82 | 1.7 |
| Accessories | 4 | 0.2 | 3 | 0.1 | 7 | 0.1 |
| Note: ^a^PSBB, Large-Scale Social Distancing | | | | | | |

Table 2. Association analysis of Internet addiction and samples' sociodemographic

| **Variables** |  | **Internet Addiction** | | **χ2** | **OR** | **95% CI** |
| --- | --- | --- | --- | --- | --- | --- |
|  |  | **Yes (n = 683)** | **No (n = 4051)** |  |  |  |
| **Gender** | Male | 382 | 2230 | 0.184 | 1 | 0.821-1.136 |
|  | Female | 301 | 1821 |  |  |  |
| **Age** | 21-40 | 634 | 3549 | 15.472*** | 1.83 | 1.349-2484 |
|  | >40 | 49 | 502 |  |  |  |
| **Education** | Up to SHS | 105 | 573 | 0.719 | 1.103 | 0.880-1.382 |
|  | Higher Education | 578 | 3478 |  |  |  |
| **Occupation** | Students | 94 | 210 | 78.48*** | - | - |
|  | Professionals | 60 | 303 |  |  |  |
|  | Office Workers/ Proprietors | 478 | 3149 |  |  |  |
|  | Civil Servants | 31 | 284 |  |  |  |
|  | Currently Unemployed | 20 | 105 |  |  |  |
| **Province of Residence** | With PSBB^a^ | 252 | 1318 | 5.014* | 1.212 | 1.024-1.435 |
|  | Without PSBB^a^ | 431 | 2733 |  |  |  |
| **Marital Status** | Single | 362 | 1377 | 90.881*** | 2.19 | 1.859-2.579 |
|  | Married/Divorced | 321 | 2674 |  |  |  |
| **Income** | Low | 35 | 165 | 11.89 | - | - |
|  | Lower-Middle | 234 | 1458 |  |  |  |
|  | Middle-Upper | 352 | 1901 |  |  |  |
|  | Upper | 62 | 527 |  |  |  |
| **Physical Distancing** | Yes | 510 | 2942 | 1.24 | 0.9 | 0.747-1.084 |
|  | No | 173 | 1109 |  |  |  |
| **COVID-19 confirmed/suspected cases within household?** | Yes | 42 | 145 | 10.174** | 1.765 | 1.240-2.513 |
|  | No | 641 | 3906 |  |  |  |
| **Perceived Internet Duration Change** | Increased | 592 | 3193 | 22.536*** | - | - |
|  | Decreased | 6 | 52 |  |  |  |
|  | Unchanged | 85 | 806 |  |  |  |
| **Daily Internet Duration During COVID-19** | 0-5 | 118 | 1086 | 46.954*** | - | - |
|  | 6-10 | 214 | 1406 |  |  |  |
|  | ≥ 11 | 351 | 1559 |  |  |  |
| **Internet Expenditure** | <250,000/17.72 USD | 22 | 108 | 0.674 | 1.215 | 0.763-1.936 |
|  | >250,000/17.72 USD | 661 | 3943 |  |  |  |
| **Age of First Internet Use** | ≤8 | 23 | 79 | 5.569* | 1.752 | 1.093-2.808 |
|  | >8 | 660 | 3972 |  |  |  |
| **Main Motives Using Internet** | Social Media | 250 | 1250 | 32.247*** | - | - |
|  | Online Gaming | 21 | 66 |  |  |  |
|  | Blogging | 0 | 1 |  |  |  |
|  | Information Seeking | 136 | 830 |  |  |  |
|  | Online Shopping | 0 | 21 |  |  |  |
|  | Entertainment | 51 | 230 |  |  |  |
|  | Cyber-relation | 1 | 2 |  |  |  |
|  | Online Gambling | 0 | 0 |  |  |  |
|  | Pornography | 1 | 4 |  |  |  |
|  | Academic/ occupational | 223 | 1647 |  |  |  |
| **Number of Social Media Apps** | 0-3 | 318 | 2436 | 44.259*** | 0.578 | 0.491-0.680 |
|  | ≥4 | 365 | 1615 |  |  |  |
| **Using Facebook** | Yes | 384 | 2241 | 0.193 | 1.037 | 0.881-1.221 |
|  | No | 299 | 1810 |  |  |  |
| **Using Instagram** | Yes | 586 | 3290 | 8.274** | 1.397 | 1.112-1.757 |
|  | No | 97 | 761 |  |  |  |
| **Using Twitter** | Yes | 284 | 1093 | 60.401*** | 1.926 | 1.630-2.277 |
|  | No | 399 | 2958 |  |  |  |
| **Using LINE** | Yes | 226 | 878 | 42.593*** | 1.787 | 1.499-2.131 |
|  | No | 457 | 3173 |  |  |  |
| **Using WhatsApp** | Yes | 647 | 3851 | 0.137 | 0.933 | 0.648-1.344 |
|  | No | 36 | 200 |  |  |  |
| **Using TikTok** | Yes | 80 | 331 | 9.250** | 1.491 | 1.151-1.932 |
|  | No | 603 | 3720 |  |  |  |
| **Using WeChat** | Yes | 8 | 56 | 0.195 | 0.846 | 0.401-1.781 |
|  | No | 675 | 3995 |  |  |  |
| **Using Telegram** | Yes | 239 | 1174 | 10.089*** | 1.319 | 1.111-1.566 |
|  | No | 444 | 2877 |  |  |  |
| **Frequent Games Genre** | MMORPG | 21 | 86 | 46.323*** | - | - |
|  | MOBA | 136 | 532 |  |  |  |
|  | FPS | 3 | 8 |  |  |  |
|  | Casual Games | 241 | 1227 |  |  |  |
|  | Do not play games | 282 | 2198 |  |  |  |
| Note: ^a^PSBB, Large-Scale Social Distancing; **p* < .05; ***p* ≤ .01; ****p* ≤ .001 | | | | | | |
